# A Viscosity Model for Blood Flows in Narrow Channels at High Reynolds Numbers Incorporating a Cell-Free Layer

**DOI:** 10.3390/mi17091030

**Published:** 2026-08-29

**Authors:** Max Lihs, Finn Knüppel, Benjamin Torner, Mario Hahne, Calvin Wolfgramm, Ang Sun, Jeanette Hussong, Frank-Hendrik Wurm

**Affiliations:** 1 Institute of Turbomachinery, Faculty for Mechanical Engineering and Ship Design, University of Rostock, 18059 Rostock, Germanybenjamin.torner@meduniwien.ac.at (B.T.); mario.hahne@uni-rostock.de (M.H.); calvin.wolfgramm@uni-rostock.de (C.W.); hendrik.wurm@uni-rostock.de (F.-H.W.); 2 Department of Cardiac and Thoracic Aortic Surgery, Medical University of Vienna, 1090 Vienna, Austria; 3 Institute for Fluid Mechanics and Aerodynamics, Technical University of Darmstadt, 64287 Darmstadt, Germany; sun@sla.tu-darmstadt.de (A.S.); hussong@sla.tu-darmstadt.de (J.H.)

**Keywords:** cell-free layer, Fåhræus–Lindqvist effect, blood flow, shear stress, particle-laden flows, microchannels, ventricular assist devices, gap flow, CFD simulations, particle migration

## Abstract

To better understand the actual flow fields in blood-contacting mechanical medical devices, such as ventricular assist devices (VADs), accurate in vitro and in silico studies are essential. Traditionally, these studies have treated blood as a single-phase fluid. In reality, however, blood is a multi-phase fluid consisting of plasma and suspended blood cells. Cell migration leads to heterogeneous cell distribution, which significantly impacts flow dynamics, particularly in the narrow gaps of these devices. This migration is not usually considered in in vitro analyses using blood analog fluids or in in silico simulations of VADs. This study presents an advanced viscosity modeling approach that accounts for cell migration effects under gap-relevant conditions. The model is based on local particle distribution data obtained from experiments with blood and particle-laden blood analog fluids in microchannels. It is applicable to both blood and particle-laden blood analog fluids, covering gap heights of 150 µm, Reynolds numbers in the range of 50 < Re < 150 and particle volume fractions of up to 30%. Previous works have investigated blood flows with significantly lower volume fractions of up to 5%. The model’s accuracy was validated by comparing results with experimental data on pressure losses of blood flowing through a microchannel, demonstrating good agreement. By incorporating variations in local viscosity, this enhanced viscosity distribution model improves the accuracy of flow simulations, offering a more realistic representation of blood flow in narrow gaps compared to the single-phase assumption. Future work will extend the model to accommodate physiological particle volume fractions of up to 45%.

## 1. Introduction

In Europe, an estimated 10 to 14 million people have heart failure, with one to two million affected by end-stage chronic heart failure [1]. When pharmacological therapy is no longer effective, heart transplantation remains the gold-standard treatment for advanced-stage heart failure. However, according to Eurotransplant, there is a critical shortage of donor hearts [1].

Ventricular assist devices (VADs) were developed as a technological alternative for treating end-stage heart failure. These pumps support a weakened heart by generating a defined pressure head at a specific blood flow rate. Most VADs are designed as turbomachines and can be categorized into two main types: axial and radial [2].

The development and optimization of modern VADs are primarily carried out through numerical flow simulations (in silico flow analyses). A key aspect of these flow analyses is predicting blood damage, which is the damage to the form and function of blood cells as they pass through the device. In this context, the computed shear stresses acting on the blood cells and their exposure times are critical factors [3]. For these blood flow analyses in VADs, the blood is often approximated as a single-phase fluid with blood-equivalent density and viscosity [4]. This assumption is valid in most regions of the VAD, as shear rates typically range within $\dot{\gamma }=150-150\times 10^{3}$ s^−1^. However, blood is inherently a multi-phase fluid, consisting of plasma and suspended blood cells, which together make up approximately 45% of the blood volume. Notably, our previous studies [5,6,7] have shown that in certain regions within VADs—particularly in narrow gaps—the single-phase assumption may not hold. To identify contributions of different groups or types of cells to the process of interest, methods like targeted cellular depletion are used in biological microfluidics, e.g., as in Ref. [8], wherein the contribution of different cell types to hepatotoxic outcomes is investigated. Since the proportion of red blood cells exceeds those of other cells in the human blood by orders of magnitude [9], it is reasonable to assume that they have the greatest influence on the rheological properties of blood and that the contributions of other cell types do not need to be considered separately for this study. Also, flow rates are much higher in this work compared to Ref. [8] ($\dot{V}\geq 13.8$ mL/min versus $\dot{V}\leq 5\;\mu$L/min).

Gaps in VADs exist because of the necessary clearance of the rotor, and are often annular gaps between concentric cylinders, with gap widths typically in the range of 40μm ≤h≤1000μm [10,11]. The pressure difference caused by the energy transferred from the rotor to the fluid causes a leakage flow through those gaps to the low-pressure side. If the gaps between concentric cylinders are sufficiently small, the effect of the curvature on the stress distribution can be neglected. In order for this small-gap assumption to be applicable, the ratio of the radius of inner cylinder $r_{i}$ (typically the rotor) and the housing $r_{o}$ must fulfill the following criterion: $r_{i}\;/\;r_{o}>0.97$ [12]. Analysis of the geometry of the several commercial VADs presented in refs. [10,11] shows that the small-gap assumption is valid for the analysis of VAD gaps. For this reason, we chose to analyze the flow in a plane channel as a valid approximation.

**Figure 1:**
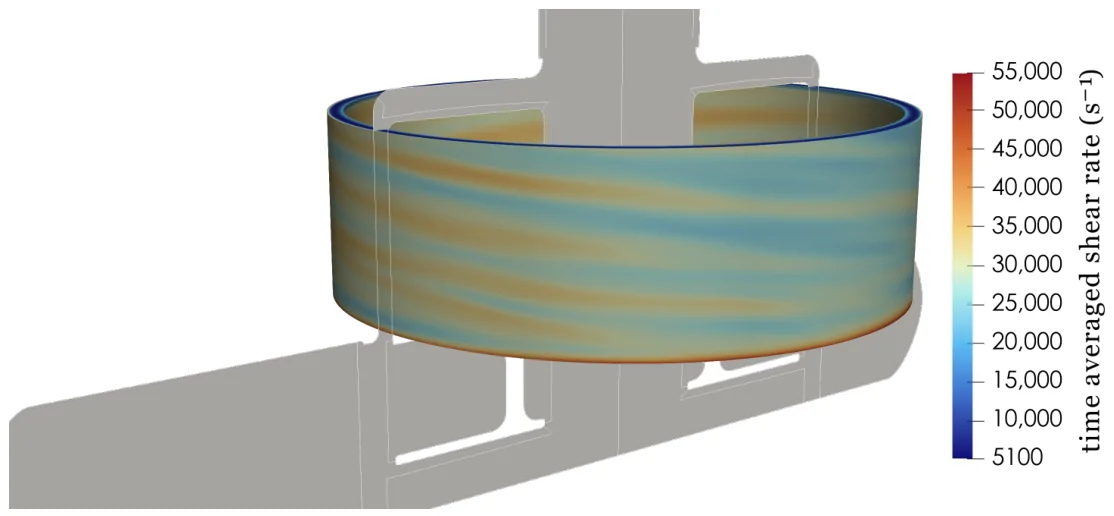
Visualization of the time-averaged shear rates in the gap between the rotor and housing of a commercial VAD. The cross section of the computational domain is shown as a 2-dimensional structure in gray.

Since gap regions also experience the highest shear rates and thus highest shear stresses in the VAD, it is crucial to accurately characterize the flow field within these gaps. Our own simulation results show that the time-averaged shear rate in these gaps is in the range of 5095 s^−1^ $\leq \dot{\gamma }\leq$ 50,445 s^−1^ (see Figure 1). The flow can be 2- or 3-dimensional, depending on the gap size and the flow rate. Also, complex flow structures like Taylor vortices can form.

In these narrow gaps of a VAD, a migration effect may occur resembling what is observed in narrow vessel flows under physiological conditions. Under physiological flow conditions ($Re=\rho \cdot c_{b}\cdot d_{h}/\mu_{rheo}$∼1), the blood cells migrate away from the wall of the vessel. This migration results in the formation of a low-viscosity cell-free layer (CFL) at the vessel wall [13,14,15,16], lowering the apparent viscosity of the blood. This is known as the Fåhræus–Lindqvist effect [17,18]. Migration effects are also known in other systems of suspended particles. Particles are often observed to migrate towards certain equilibrium positions, which is known as inertial focusing. The position of these focusing points is influenced by the shape of the channel as well as the properties of the particles and the Reynolds number of the flow [19]. A similar effect is proposed to occur in the narrow VAD gaps. However, in contrast to vessel flow, flow in a VAD gap typically has a significantly higher Reynolds number (Re∼100), which will influence the cell migration process. In both scenarios (vessel and VAD gap), the change in viscosity affects the blood flow field, leading to generally lower shear stresses and pressure drops compared to a homogeneous cell distribution [20].

**Figure 2:**
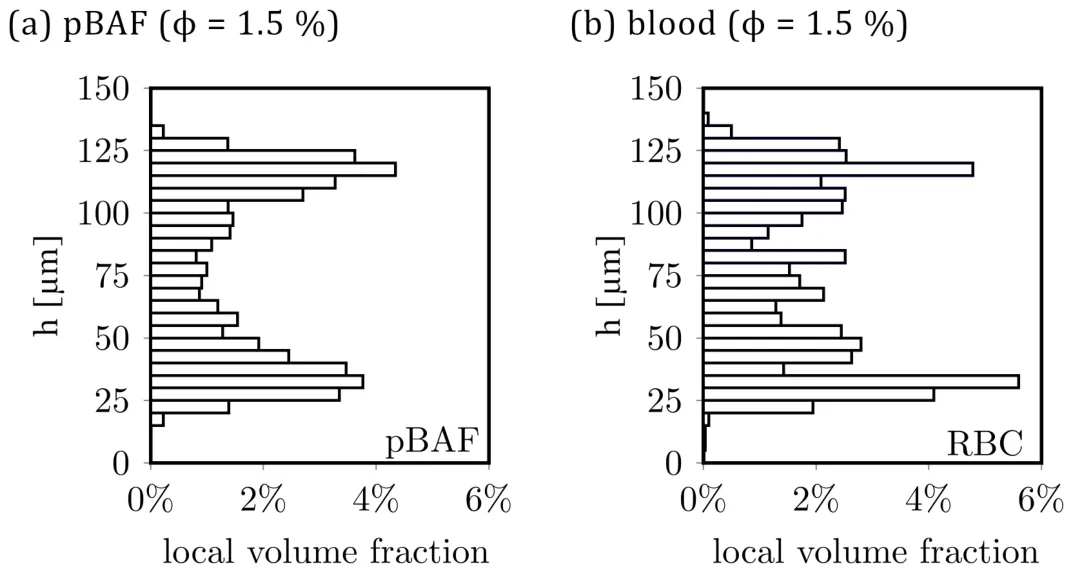
(**a**) Height-dependent distribution of the volume fraction of pBAF (ϕ=1.5%) in a microchannel at Re =150. (**b**) Distribution of the local volume fraction of blood (ϕ=1.5%) in the same microchannel at Re=150 [7].

Our research group has already investigated this topic in refs. [5,6], with a particular focus on experimental analysis of the migration effects in blood and particle-laden blood analog fluids (pBAFs) at low particle volume concentrations (ϕ<5%). The use of pBAFs offers important advantages over the use of real blood, such as better handling [21], less agglomeration [22] and, most importantly, better optical accessibility [22,23]. In a further study, we showed that at low hematocrits of 1.5% and a Reynolds number of 150, the local distribution of blood behaves very similarly to the pBAF (see Figure 2), taken from our previous publication [7]. Rigid particles in microchannel flow experience forces due to inertial effects. The dominating forces in planar channel flow are known as the Wall effect and the shear-induced lift [24]. In addition, deformable particles experience deformation-induced lift, caused by the change in the shape of the particle when exposed to shear stresses of the flow [25,26]. This additional force influences the migration behavior by counteracting the forces pushing the particles towards the wall. However, the experimental results presented in refs. [6,7] show comparable particle distribution and CFL formation in blood and pBAF. This might be due to factors unaccounted for in Ref. [25], such as particle interaction or the square-shaped cross section of the channel used in refs. [6,7].

To define the CFL height, the criterion $\phi_{loc}\leq 0.1\cdot \phi$ was applied, where $\phi_{loc}$ represents the local particle volume fraction in the near-wall region and ϕ denotes the bulk volume fraction. This criterion has proven to be a reliable way to identify the CFL in combination with the method of counting detected particles in bins of Δh=5 μm, which corresponds to the precision with which the position of the cells and particles was identified, as we describe in Ref. [7].

A viscosity distribution model was developed that addresses the effect of the particle migration by modeling the viscosity change over the height of the geometry (vessel, channel, VAD gap) at Reynolds numbers higher than physiological ones [20]. The viscosity distribution model was validated with blood analog fluids and blood up to a particle volume fraction of 5%. The objective of this study is to extend and refine the viscosity distribution model for higher volume fractions of up to 30%. To achieve this, pressure loss measurements in the blood flow in microchannels were conducted up to a volume fraction of 30%. Additionally, the viscosity distribution model from Ref. [6] was enhanced by employing the results of our viscosity measurements for blood at different volume fractions.

## 2. Materials and Methods

### 2.1. Experimental Setup for Analyzing the Impact of Cell Migration in Narrow Gaps

For the experimental investigations, two identical test benches were utilized—one at the University of Rostock and the other at Technical University of Darmstadt. At the Technical University of Darmstadt, optical measurements were conducted using Astigmatism Particle Tracking Velocimetry (APTV) to analyze the CFL height and the local distribution of particles and cells. Meanwhile, at the University of Rostock, the focus was on assessing the impact of the CFL on flow dynamics, specifically examining how particle migration influences wall shear stress (WSS) and the pressure drop across the channel.

**Figure 3:**
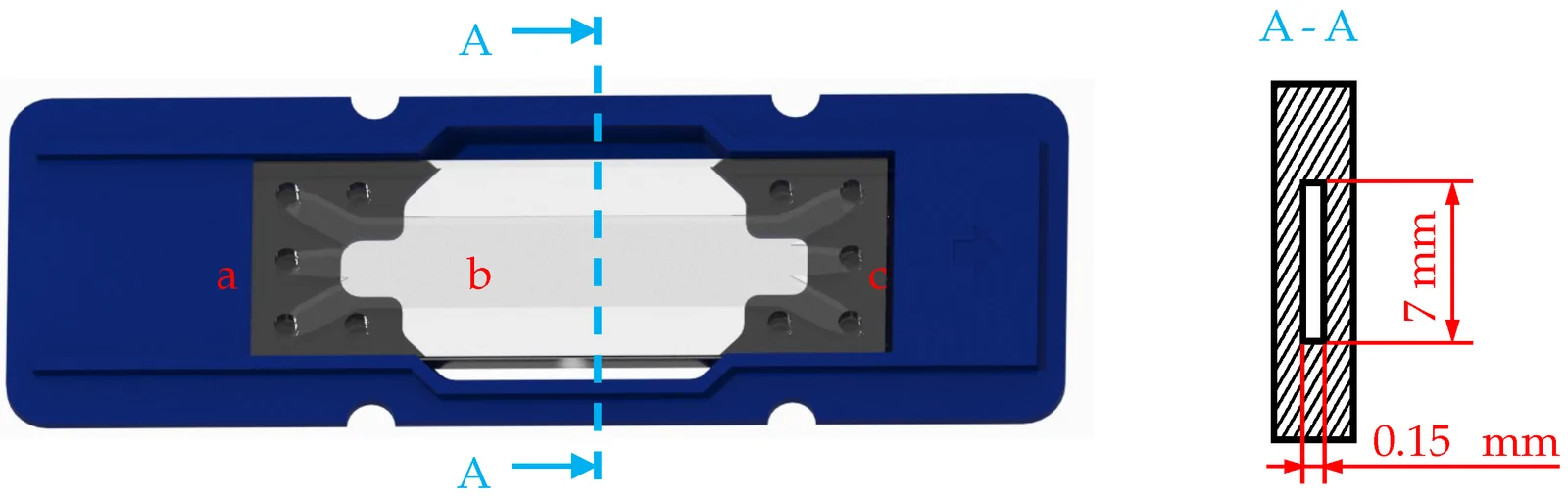
(**Left**): Schematic illustration of the used microchannel. (a): Inlet openings. (b): Straight channel section with rectangular cross section. (c): Outlet openings. (**Right**): Sketch of the channel cross section.

The sister test benches are described in detail in Ref. [5] and will be briefly outlined here. Each setup consists of a syringe pump, hose connections, a microchannel, and a reservoir. The microchannels in the test bench were designed as a generic test case to investigate cell and particle migration, the subsequent formation of the cell-free layer (CFL), and the influence of the microfluidic environment under flow conditions similar to those in VAD gap flow. Rectangular channels with an aspect ratio greater than 15 were used to ensure a quasi-planar channel flow [27], as shown in Figure 3. This design is also referred to as the Hele-Shaw cell and is commonly used for investigating microfluidic particle-laden flows; see for example Refs. [28,29,30]. Because of the aspect ratio of the channel’s cross section, the flow can be assumed to be planar and the influence of the side walls (in Figure 3, right side: walls with the height of 0.15 mm) can be neglected. Capillary tubes are connected to the inlet and outlet of the channel via a Fluidic Connect Pro chipholder manufactured by Micronit Microfluidics BV (Enschede, the Netherlands).

Reynolds numbers were investigated in the microchannels within the range of 50<Re<150, which represents typical values found in VAD gaps [3]. The Reynolds number is determined by the bulk velocity $c_{b}$ in the microchannel, which is the average velocity in the channel’s cross section $c_{b}=\dot{V}/A$, and the hydraulic diameter of the plane channel $D_{h}=2h$, where *h* is the channel height. Additionally, the density ρ and dynamic bulk viscosity $\mu_{rheo}$ play a crucial role in these calculations. The values of $\mu_{rheo}$ are measured using a rheometer.

(1) $$Re=\frac{\rho \cdot c_{b}\cdot D_{h}}{\mu_{rheo}}$$

**Figure 4:**
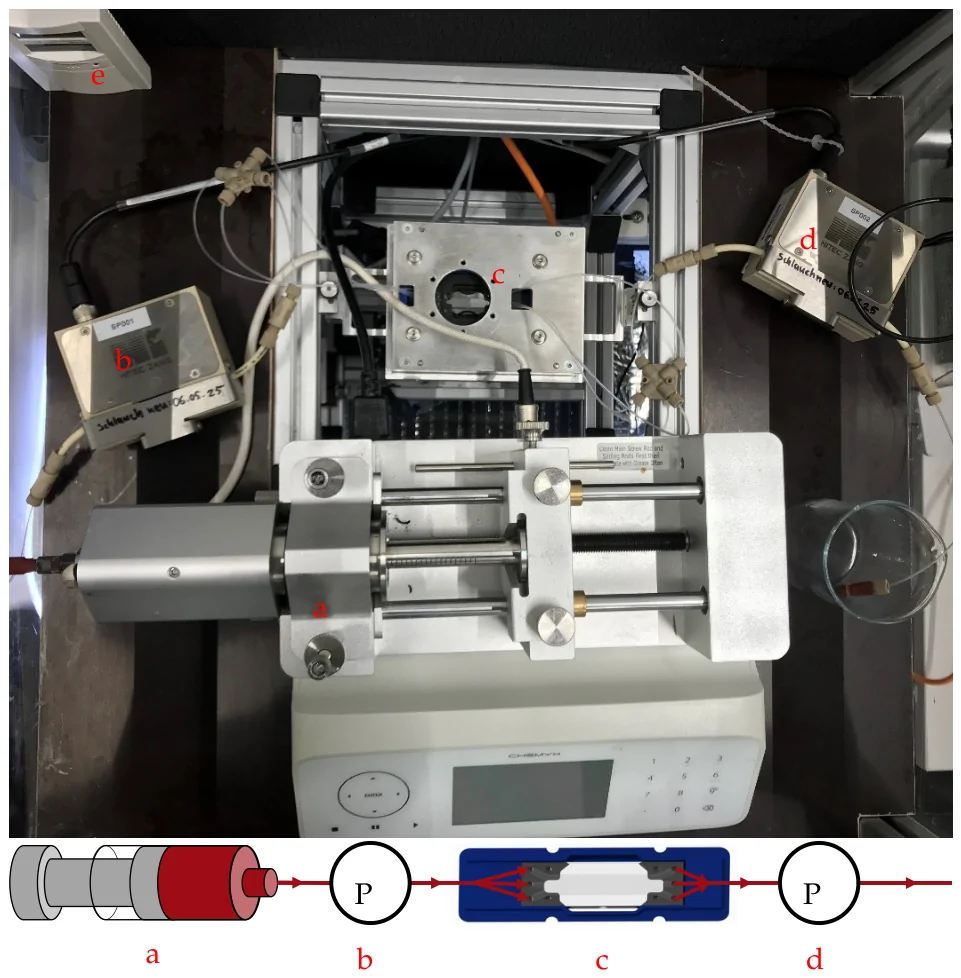
Test bench for the pressure (loss) measurements in the microchannels with the following components: (a) syringe pump with 100 mL syringe, (b) pressure sensor 1, (c) microchannel in chip holder assembly, (d) pressure sensor 2, and (e) digital thermometer. (**Top**): Photograph from above. (**Bottom**): Schematic.

For volume fractions of up to 20%, particle migration was measured directly by APTV to optically detect the CFL formation and CFL height in the microchannel. Using an astigmatic defocusing lens arrangement, the position of a particle can be determined by its optical distortion on the image captured on a camera. An image-processing algorithm is then used to group and count the detected particles by their position on the channel height. For more details on the APTV procedure, refer to Ref. [7]. Also, the formation of a CFL due to particle migration results in reduced wall shear stresses, as only low-viscosity plasma flows near the walls. This reduction in shear stress also decreases the pressure losses in the microchannels (compared to a fluid with a homogeneous density ρ and viscosity $\mu_{rheo}$ distribution). Therefore, we intended to measure the pressure losses within the particle-laden microchannel flows. The pressure on the inlet and outlet sides of the microchannel was measured with IS-P-Tube pressure transmitters by HiTec Zang GmbH, Herzogenrath, Germany with a range of $\left(0\leq p\leq 20\right)\times 10^{5}$ Pa and a precision of $\pm 18\times 10^{2}$ Pa. Further details on the measurement procedures can be found in Ref. [5]. The test bench is shown in Figure 4.

#### 2.1.1. Blood: Collection and Preparation and Blood Analog Fluids

Fresh porcine blood, obtained from the Research Institute for Farm Animal Biology (FBN), was treated with citrate phosphate dextrose adenine (CPDA-1) as an anticoagulant. The hematocrit of the pig blood was determined at the Institute for Clinical Chemistry and Laboratory Medicine, University Hospital Rostock. Part of the blood sample was centrifuged to separate the plasma from the blood cells and to adjust the hematocrit to the desired value (up to 30%). Densities and viscosities of the respective blood samples were measured using a rheometer AntonPaar MCR302e (Anton Paar Group AG, Graz, Austria) and density meter DMA 5000M (Anton Paar Group AG, Graz, Austria), respectively.

For the APTV measurements, plasma was replaced with phosphate-buffered saline (PBS) as the carrier liquid. To ensure a homogeneous PBS solution, multiple centrifugation steps were performed, followed by refilling with fresh PBS as part of a washing process. Erythrocytes were stained with the fluorescent dyes rhodamine and rhodamine B. A small fraction of blood (1.5% of the cell fraction mixed with the dye) was added to the hematocrit samples to enable optical measurement of the set hematocrit level. Table 1 shows the average density ρ and viscosity μ of the blood preparation. This was subsequently used to set the Reynolds number for the experimental investigations and for the numerical simulation. It is noticeable in Table 1 that the density and viscosity decrease significantly with the carrier liquid PBS (APTV measurement). This is due to the fact that the carrier fluid, namely PBS, has a lower density than the plasma ($\rho_{plasma}=1020.4$ kg/m^3^ compared to $\rho_{PBS}=1000.36$ kg/m^3^) [31]. The preparation and formulation of the particle-laden blood analog fluids (pBAFs) for the experiments are detailed in Ref. [5].

#### 2.1.2. Effect of Carrier Fluid Viscosity on Inertial Particle Migration

Based on the fluid properties presented previously, the following section briefly discusses the influence of the viscosity of the carrier fluid on inertial particle migration, with particular emphasis on conditions of constant bulk Reynolds number. The particle Reynolds number $Re_{p}$ is introduced here as a characterization of the lift forces acting on a particle. It is defined from the channel Reynolds number (Equation (1)), the particle diameter $a_{p}$ and the channel height *H*.

(2) $$Re_{p}=Re\cdot {\left(\frac{a_{p}}{H}\right)}^{2}$$

It can be assumed that variations in the carrier fluid (plasma/PBS) do not substantially alter inertial migration behavior at the investigated Reynolds numbers [32]. However, the investigated parameters only partly overlap with the experiments in this study. To ensure similar migration behavior, the flow rate for the experiment was set to achieve the Reynolds number under investigation. Therefore, the bulk Reynolds number was kept constant between experiments with blood and PBS. Refs. [33,34] suggest that the equilibrium position and migration velocity of suspended particles depend on the blockage ratio $a_{p}\;/\;H$. This ratio was kept constant over the experiments by selecting beads with a diameter equal to the characteristic diameter of RBCs (7.5 μm). Channel height *H* was constant over all experiments. With Re and $Re_{p}$ matched, the migration behavior over all experiments could be assumed to only vary with the flow parameters. Bhagat et al. report that a lower threshold for observable migration effects exists at approximately $Re_{p}$∼0.05 [35]. In our tests, the particle Reynolds number was $Re_{p}=0.375$.

### 2.2. Numerical Setup for the Simulations with the Zonal Viscosity Distribution Model

#### Numerical Setup for the Simulations

**Figure 5:**
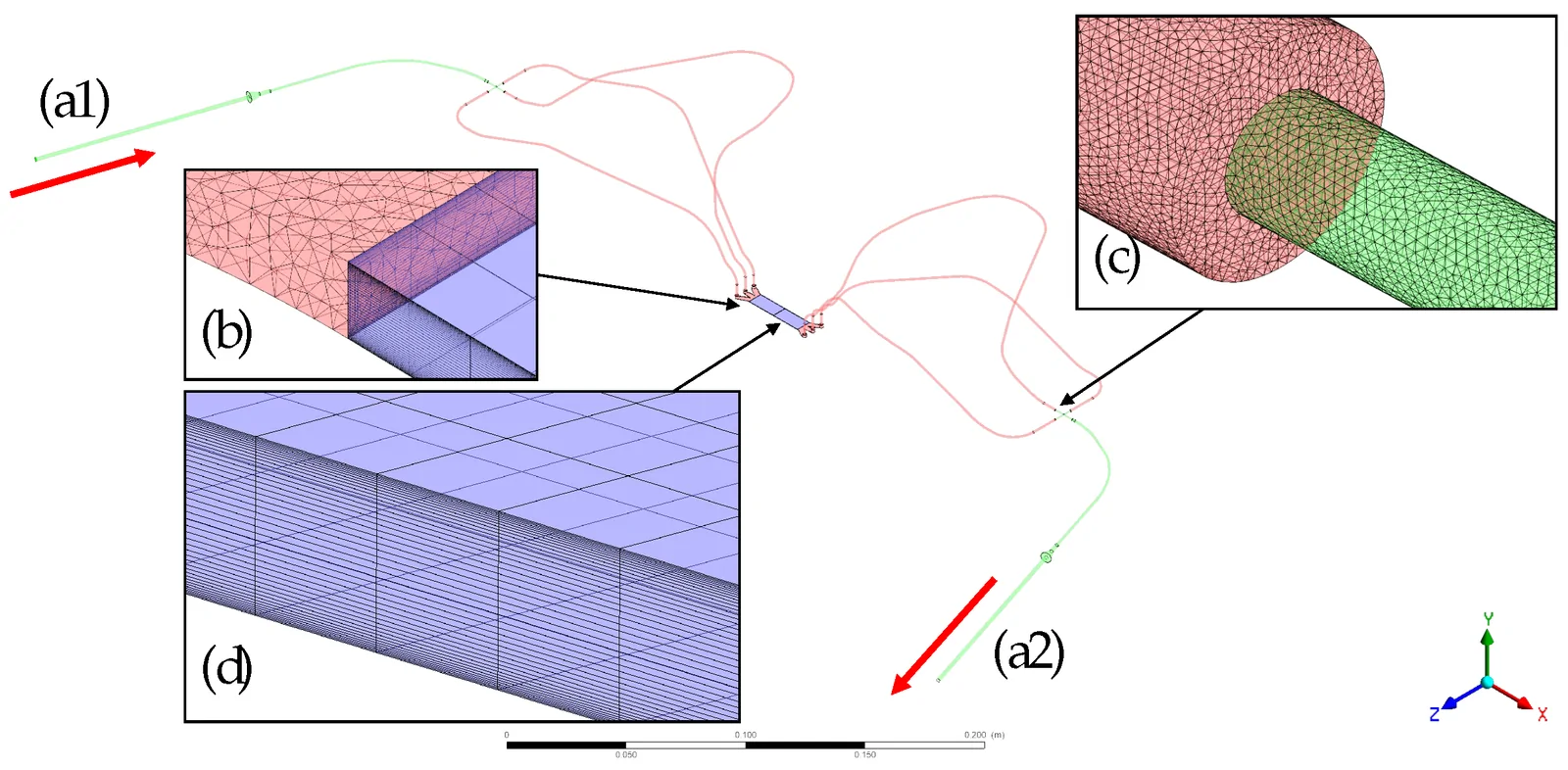
Computational domain for simulations of the test rig between the two pressure sensors (**a1**,**a2**). (**b**): Detail of the interface between the inflow area (red, unstructured mesh) and the straight channel section with a rectangular cross section (blue, structured mesh). (**c**): Exemplary detail of the unstructured mesh used for the tubing. (**d**): Detail of the structured mesh used for the straight channel section with a rectangular cross section.

A digital mock-up of the test bench was used for the simulation setup, based on a previous version described in Ref. [5]. In this model, the test bench was digitally replicated, allowing the simulation to operate in the whole test bench, including the two pressure sensors (see Figure 5).

Furthermore, a viscosity distribution model was developed in Ref. [6] to numerically simulate the effect of particle migration within the channel flow. In earlier works, this model had only been used to simulate flows with volume fractions of up to ϕ=5%.

The simulation domain has now also been extended to account for the entire test bench. Particle migration effects are considered only in the microchannel while assuming a homogeneous particle distribution in the rest of the test bench. Migration does not occur there or is negligible. To account for the effects of the particle migration, the domain of the microchannel is split in the wall-normal direction in two zones by applying a step function: the near-wall CFL region with reduced viscosity and the core region with higher viscosity. A comparison is made between the experimental pressure measurement results and the simulated ones, obtained using this approach, up to volume fractions of ϕ=30%.

In order to compare and evaluate the different fluids, in our study, we used a dimensionless pressure coefficient $c_{p}$. It is determined via the following equation:

(3) $$c_{p}=\frac{\Delta p}{\frac{1}{2}\cdot \rho \cdot c_{b}^{2}}$$

By comparing the pressure coefficients, it is possible to indirectly determine the influence of the cell-free layer in the plane microchannel. For single-phase fluids, the ratio $c_{p}/c_{p,0\%}$ is independent of density and viscosity and remains constant for a certain Reynolds number. It is equal to one. For a multi-phase fluid with a homogeneous particle distribution, the ratio also remains one. As a cell-free layer forms (inhomogeneous distribution), the relative pressure loss decreases due to the decreased bulk viscosity and the ratio $c_{p}/c_{p,0\%}$ becomes less than one. In this way, the trends in the reduction in pressure loss in particle-laden flows with the formation of a cell-free layer can be determined as a function of the volume fraction and Reynolds number.

Laminar flow simulations of the test bench were performed for Reynolds numbers in the range of 50≤Re≤150. The simulations were performed by solving the Navier–Stokes equations in ANSYS CFX 2022R2 (Ansys Inc., Pittsburgh, PA, USA) with a finite volume method. The computational domain of the simulation with the used computational grid is shown in Figure 5.

The domain shown in Figure 5 represents the test bench, extending from the inlet pressure sensor (a1) through the microchannel to the outlet pressure sensor (a2). The grid contains ≈23 million nodes with angles ≥32° and aspect ratios ≤69. The whole periphery and inlet section of the microchannel were meshed using tetrahedral elements (with five prism layers near the walls). Here, the fluid is treated as a single-phase fluid with an averaged, uniform density and viscosity. The straight section of the channel was meshed using hexahedral elements. In this part of the domain, the local viscosity distribution was modeled using the viscosity distribution model, taking into account the multi-phase nature of blood flow. In the CFL, we assumed the plasma viscosity. Outside of the CFL, the erythrocytes are present, which leads to a higher local viscosity. In the viscosity distribution model, this was implemented by a step function with high viscosity, so that the height-averaged viscosity correlates with the viscosity measured by a rheometer at a specific hematocrit. This is explained in detail in Ref. [6].

To highlight the limitations of assuming a single-phase flow throughout the whole test bench, we also performed simulations using a constant bulk viscosity. These results will be compared with both the CFD simulations incorporating multi-phase effects (via the viscosity distribution model) and the experimental data, providing a comprehensive evaluation of the viscosity distribution model’s performance.

## 3. Results and Discussion

### 3.1. Expanding the Viscosity Distribution Model for Simulating Blood Flow in the Test Bench

In a previous study by Knüppel et al., a viscosity distribution model was developed that applies to pBAF and blood with a volume fraction of up to 5% [20]. The model employs the Einstein–Roscoe equation to characterize the viscosity changes induced by particle migration:

(4) $$\mathrm{Einstein}–\mathrm{Roscoe}:\;\mu_{loc}\left(h\right)=\mu_{plasma}\cdot {\left(1-1.35\cdot \phi_{loc}\left(h\right)\right)}^{-2.5}$$

In Equation (4), the factor of 1.35 represents the theoretical maximum concentration in suspensions of spherical particles [36]. An adaptation of this factor to more accurately describe blood, where cells have a different maximum concentration due to their deformability, was proposed in reference [37]. The exponent of −2.5 was first introduced in Ref. [36] to expand the original Einstein equation to higher particle concentrations.

**Figure 6:**
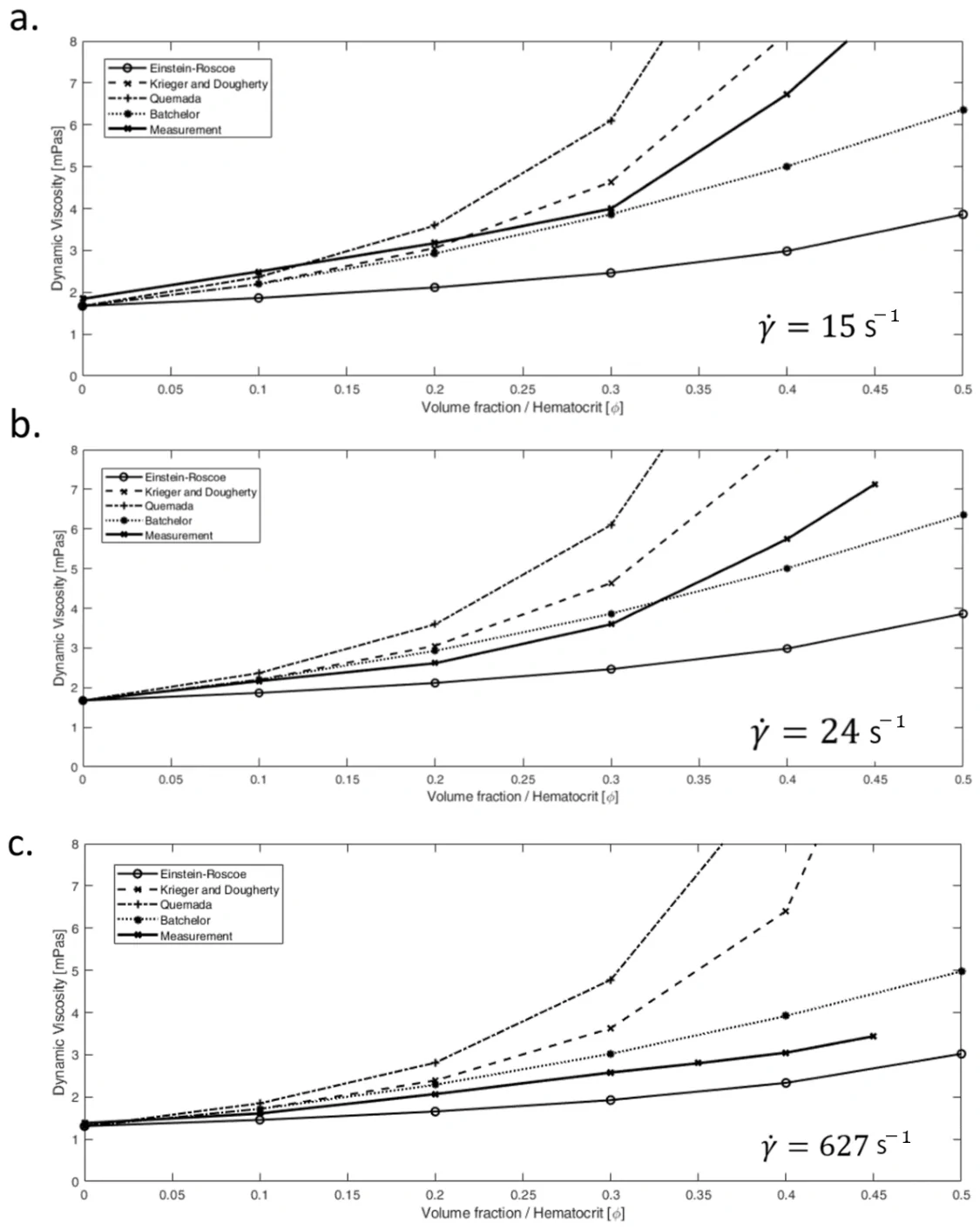
Comparison of the common viscosity models for blood at shear rates of $\dot{\gamma }=15\;s^{-1}$ (**a**), $24\;s^{-1}$ (**b**), and 627 s^−1^ (**c**).

In the present study, viscosity measurements were conducted over a range of shear rates ($15\;s^{-1}\leq \dot{\gamma }\leq 627\;\;s^{-1}$) in order to identify a suitable viscosity model. For this purpose, commonly used viscosity models such as the Quemada, Krieger–Dougherty and Batchelor equations were applied for shear rates of $\left(15,24,627\right)$ s^−1^ (see Figure 6).

The results of the different shear rates show that some viscosity models are in good agreement at low shear rates ($\dot{\gamma }\approx 15\;s^{-1}$), as shown in Figure 6. At the highest shear rate of $627\;s^{-1}$, the Einstein–Roscoe model shows the best fit to the measurement results.

None of the applied viscosity models capture the rheological behavior of blood at all shear rates, despite modeling its shear-thinning behavior. Due to this fact, the measurement data were used in the developed viscosity distribution model. A polynomial fit was applied to the measured data, providing an accurate representation of the viscosity change caused by the variation in the hematocrit across the channel. 

(5) $$\mu =\left(a\cdot \phi^{2}+b\cdot \phi +c\right)\cdot 1\;Pa\cdot s$$

The coefficients in Equation (5) are as follows: $a=6.334\times 10^{-3},\;b=2.037\times 10^{-3},\;c=1.358\times 10^{-3}$. A representative shear rate of $627\;s^{-1}$ was selected for the numerical modeling. This decision was based on the observation that no significant changes in the dynamic viscosity occurred above this value [38]. In all experiments conducted for this study, the value of the shear rate, averaged over the channel height, well exceeded this threshold. The minimum average shear rate of 4323.8 s^−1^ in the microchannel occurred when using pure plasma (HCT =0%) at a Reynolds number of Re=50. The highest average shear rate of $24.92\times 10^{3}$ s^−1^ occurred at measurements with blood with HCT =30% at Re=150. In addition, it was observed in the experiments with microchannels that no significant difference in lateral particle migration occurred at Reynolds numbers in the range of 50 to 150 [5,33].

### 3.2. Characterization of the Particle/Cell Distributions in pBAF and Blood Flow

To accurately parameterize the viscosity distribution model for higher hematocrits as well, optical measurements of the cell-free layer (CFL) height were performed. Previous studies [5] have demonstrated that the CFL height remains relatively stable across the investigated Reynolds number range (50<Re<150). Based on these findings, experiments were conducted at a fixed Reynolds number of Re=150 to ensure consistency.

**Figure 7:**
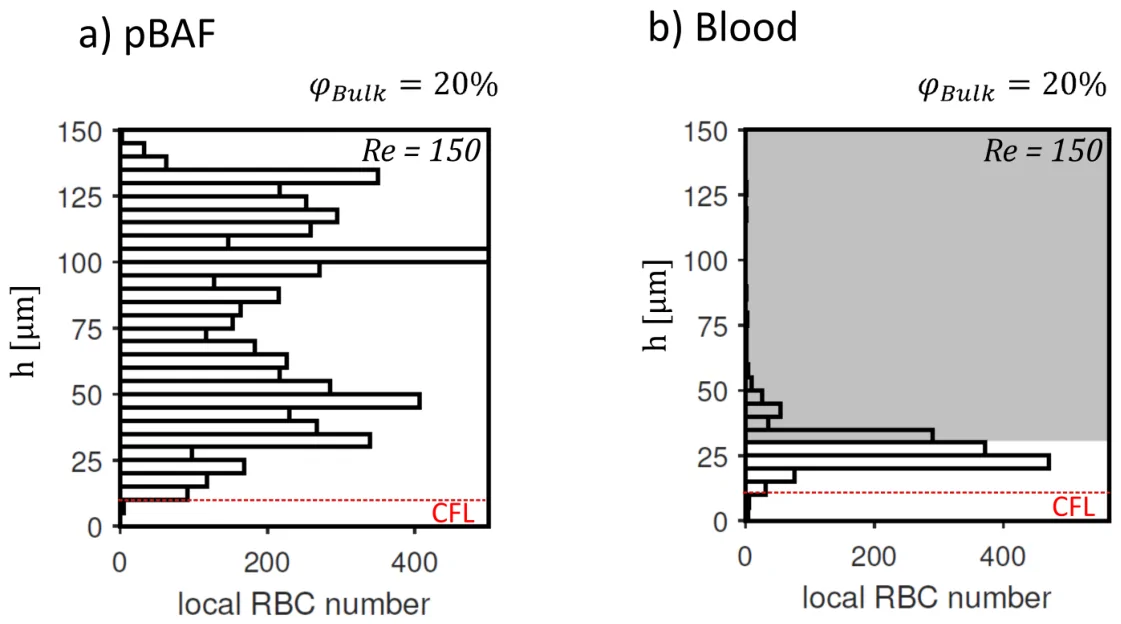
Local distribution of red blood cells (RBCs) and particles in (**a**) pBAF and (**b**) blood at ϕ=20% and Re=150. The red dashed line indicates the CFL boundary, which is 12.5 µm. The gray area marks poor optical accessibility (h > 30 µm).

Figure 7 shows the local distribution of particles and red blood cells (RBCs) for both pBAF and blood at a volume fraction/hematocrit of 20%. In both cases, a defined CFL near the channel walls is observed. The use of pBAF facilitates improved optical accessibility [38,39]. In contrast, quantifying local RBC distributions in blood presents greater challenges due to the light-absorbing characteristics of hemoglobin, which hinder optical measurements at distances from the channel wall greater than 30 µm [7]. Therefore, the local distribution of RBCs cannot be accurately determined in this area. Here, cells cannot be detected reliably or cannot be detected at all. Because of this, in Figure 7, fewer cells are visible for blood than for pBAF. Nonetheless, the CFL height can still be determined with sufficient reliability in both fluids.

The CFL height was determined to be approximately 12.5 µm for both investigated fluids. This experimentally determined CFL height is used in the employed viscosity distribution model. A key observation is that the CFL height is very similar for both fluids at a volume fraction of ϕ=20% (see Figure 7).

### 3.3. Flow Simulations and Analysis of the CFL Impact on the Particle-Laden Flow

**Figure 8:**
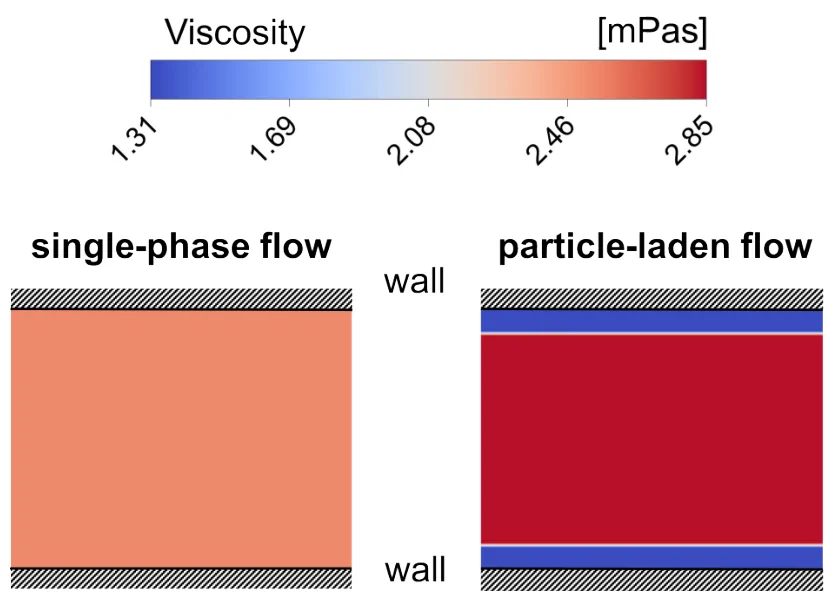
(**Left**): Local viscosity distribution in the microchannel for a single-phase flow. (**Right**): A particle-laden flow with the viscosity distribution model. The viscosity varies in the CFD with the viscosity distribution model, showing lower values in the CFL region and increasing towards the core flow.

The viscosity distribution model is based on the measured CFL height. The viscosity distribution model reveals distinct local variations: in the CFL region, viscosity is significantly lower due to the absence of suspended cells, while outside the CFL, it increases abruptly before reaching a plateau. Assuming blood as a single-phase fluid, the viscosity remains constant across the entire channel height. The comparison of the viscosity distribution over the channel height with a red blood cell volume fraction ϕ of 30% for the single-phase model and the viscosity distribution model is shown in Figure 8.

**Figure 9:**
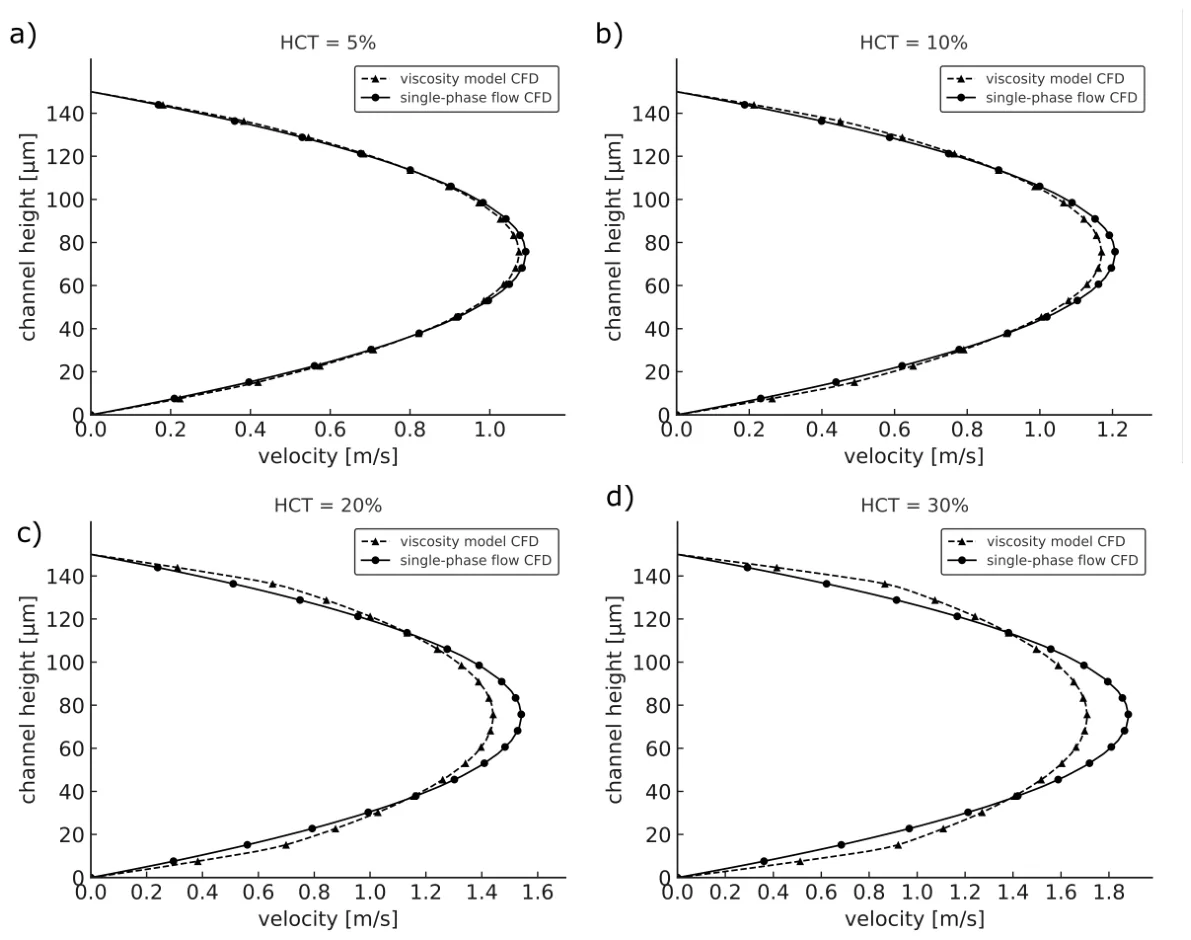
Comparison of simulated blood velocity profiles in the microchannel at Re = 150 and (**a**) ϕ=5%, (**b**) ϕ=10%, (**c**) ϕ=20% and (**d**) ϕ=30%: CFD considering a single-phase flow (constant viscosity) and CFD considering multi-phase effects (viscosity distribution model).

Despite not completely modeling the actual distribution of cell concentration over the channel height, the difference between employing the step function and employing a polynomial fit of the cell concentration amounted to a deviation of <3% in calculated bulk viscosity. Since the gradient of viscosity over *h* is very steep at the CFL boundary, this simplification is justified. The viscosity variation directly influences the velocity profile. This is especially the case for the velocity distribution over the channel height. In Figure 9a, the velocity profiles over the channel height for the CFD single-phase fluid assumption and the CFD viscosity distribution model are compared. The volume fraction ϕ for red blood cells corresponds to 5%. In the single-phase assumption, the velocity profile follows a nearly parabolic shape, with higher maximum velocities. However, in the viscosity distribution model, the presence of a CFL leads to a reduction in peak velocity and a redistribution of shear stresses. This effect becomes more pronounced with increasing hematocrit levels ϕ. See Figure 9b for a volume fraction ϕ of 10%, (c) for 20% and (d) for 30%. This increase is caused by the increased local viscosity outside the CFL.

The reduction in the peak velocity by using the viscosity distribution model impacts the velocity distribution in the direction of channel height, resulting in slightly higher velocity gradients in the near-wall region. The wall shear stress is a function of the velocity gradient in the channel height direction and the viscosity. In Table 2, the wall shear stress is shown for the single-phase fluid and the viscosity distribution model, calculated from the CFD results. Wall shear stress measurements were previously conducted by us for volume fractions up to 5% and Reynolds numbers up to 150 [6]. The wall shear measurements were conducted using the wall shear stress sensor RealShear M-Series, Lenterra Inc., Newark, NJ, USA. The measurement principle is based on the deflection of a plate by the wall shear stress acting on it. Higher hematocrit values lead to a faster agglomeration of cells in the gap between plate and housing, eventually blocking the gap and impairing the measurement. For that reason, WSS measurements for higher hematocrits cannot be presented in this paper.

In addition, the analytical values of the wall shear stress are calculated using the following equation for a single-phase fluid:

(6) $$\tau_{w}=\frac{\lambda \cdot \rho \cdot c_{b}^{2}}{8};\;with\;\lambda =\frac{96}{Re}$$

The results of the single-phase fluid CFD simulations for the wall shear stress show good agreement with the corresponding analytical solutions [40]. The deviation between the single-phase CFD and the analytical model remains consistent across different volume fractions ϕ. However, it becomes evident that the wall shear stress predicted by the CFD simulations using the viscosity distribution model is systematically lower than that of the single-phase fluid CFD across all values of ϕ. The wall shear stress obtained from the viscosity distribution model increases significantly with increasing volume fraction ϕ. This trend is particularly important in high-shear regions such as in the rotor of a VAD, where the single-phase flow simulations may substantially overestimate the wall shear stress. This overestimation may be attributed to the neglect of particle migration effects in the single-phase CFD approach. In the CFL, the viscosity is equal to the viscosity of the carrier fluid $\mu_{\mathrm{carrier}}$. The resulting local shear stresses can be calculated using Equation (7):

(7) $$\tau_{ij}{|}_{CFL}=\mu_{\mathrm{carrier}}\cdot \left(\frac{\partial u_{i}}{\partial x_{j}}+\frac{\partial u_{j}}{\partial x_{i}}\right)$$

**Figure 10:**
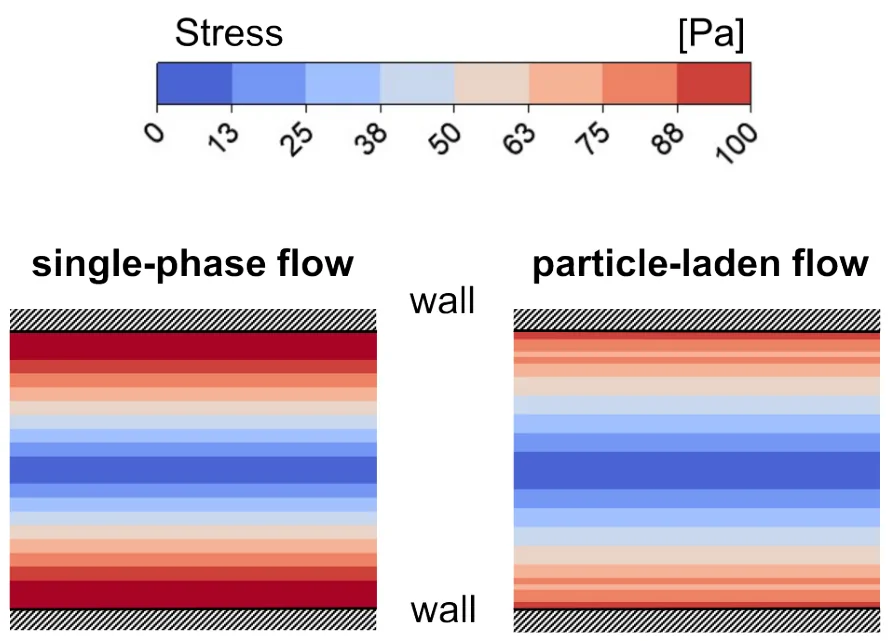
Shear stress distribution in the center of the microchannel for a volume fraction ϕ of 30% with single-phase fluid and particle-laden fluid.

The impact of particle migration and CFL development on the stress field in particle-laden flows is illustrated in Figure 10. The shear stress distribution is shown for the volume fraction of ϕ=30%. The value of the stress is reduced by ≈20% at the wall. Due to CFL formation, RBCs are not present in the regions that experience the highest stresses. With the single-phase assumption, stresses τ≥100 Pa occur near the wall while cells at the CFL boundary experience maximum stresses of up to 88 Pa according to the simulation. In both cases, the stress is lower than the threshold for hemolysis of 150 Pa [41]. However, the difference is still relevant, because lower stresses of τ≥30 Pa can already impair the deformability of human RBCs [42]. Since human and porcine blood, which was used for this study, exhibit very similar hemolysis in the range of 25 Pa ≤τ≤320 Pa [43], it is reasonable to assume that a comparable phenomenon occurs for porcine RBCs.

**Figure 11:**
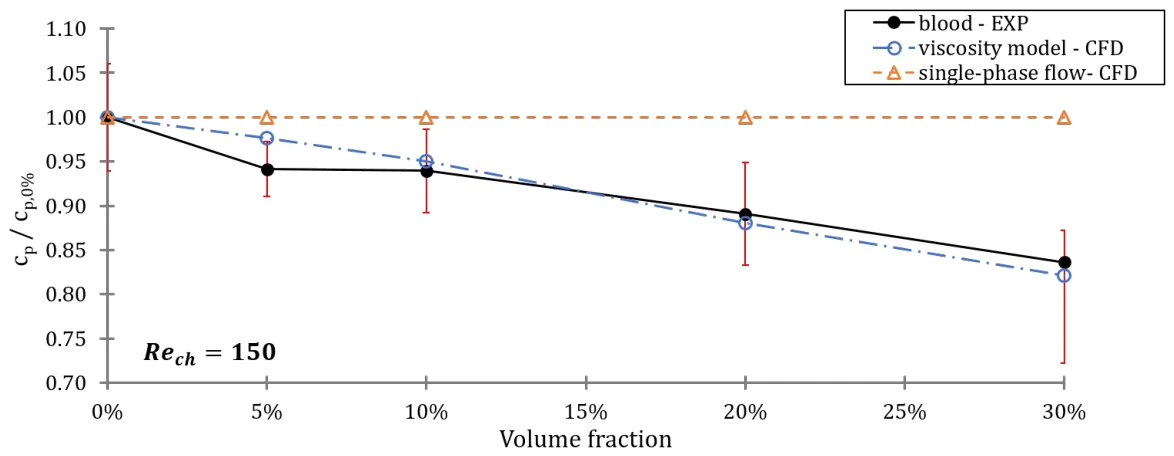
Comparison of normalized pressure loss coefficient $c_{p}/c_{p,0\%}$ in the microchannel blood flow at the Reynolds number Re = 150 as a function of volume fraction ϕ. The experimental data (solid black) is compared to the CFD with the single-phase assumption (dashed orange) and the CFD using the viscosity distribution model (dash-dot blue).

An aspect of this study was to evaluate the effect of particle migration on the pressure losses in blood flows. Figure 11 presents a comparison of the normalized pressure loss coefficient $c_{p}/c_{p,0\%}$ for Reynolds number (Re = 150) at different volume fractions ϕ. Experimental data (solid black) are compared to simulations based on the single-phase flow assumption (dashed orange) and particle migration effects by the viscosity distribution model (double dashed blue).

The results indicate that the CFD with the single-phase flow assumption systematically overestimates pressure losses for increasing particle volume fractions. In contrast, the viscosity distribution model shows a consistent agreement with the experimental measurements. Specifically, the CFD results obtained using the viscosity distribution model fall within the error bars of the experimental data, confirming its capability in predicting the pressure losses in the blood flow. On the other hand, the CFD results based on the single-phase flow assumption significantly deviate from the experimental data. These results highlight the necessity of incorporating cell migration effects into viscosity distribution models when simulating blood flow in narrow gaps. The improved viscosity distribution model provides a more accurate representation of local shear stresses and pressure losses, which are crucial for predicting the hemocompatibility of ventricular assist devices (VADs).

While other methods for simulating particle-laden flows are available—for example, a model suitable for deformable particles, developed by Polykarpou et al. [44]—the proposed model has some advantages that make it better suited for use during early design phases. Two-fluid approaches (also referred to as Eulerian–Eulerian Models), as well as Euler–Lagrangian approaches, require additional equations to be solved and incorporate coefficients to model the influence of the particles on the behavior of the fluid [45,46]. Those can be hard to model for red blood cells because of their complex mechanical behavior. Due to the high number of RBCs per mm^3^—typical values are in the range of $4.6\times 10^{6}$ to $6.1\times 10^{6}$ cells per mm^3^ for adults [47]—particle-resolved simulations require enormous amounts of computational resources for applications at macroscopic scales [48] that are often prohibitive. These drawbacks make them unsuitable for simulations of VADs, where requirements for the resolution of computational meshes are high. The proposed model can be implemented specifically in relevant regions of the domain, saving computational resources. It also offers an opportunity to compare results obtained with other methods of simulating particle-laden flows to results closely related to experimental observations with blood.

## 4. Limitations

The viscosity distribution model represents a significant improvement for simulating blood flow in narrow gaps. However, its applicability is currently limited as the model has only been validated for the flow in narrow gaps with a gap height of 150 µm and particle volume fractions of up to 30%. The physiological hematocrit values are in the range of 37–50%.

The local viscosity distribution defined by the step function generates a numerical error in the stress field, as shown in Figure 10. Since modeling was done for a representative shear rate of $\dot{\gamma }=627$ s^−1^, shear-thinning effects are not modeled. Thus, the proposed viscosity distribution model is only applicable at high enough shear rates ($\dot{\gamma }\geq 627$ s^−1^). Despite the fact that VAD gaps can usually be approximated to be sufficiently small for the curvature to be negligible (see Section 1) for the stress distribution, the transferability to curved geometries might be limited due to effects on particle migration that only occur in a curved channel. Also, the influence of superimposed rotation on the migration behavior of red blood cells still needs to be investigated.

The next step in our research is to extend the model to higher volume fractions. To this end, we are planning further experiments using blood with volume fractions of up to 45%. APTV measurements, which are currently undertaken as part of this project, will allow for a more detailed validation of the proposed model and a direct comparison of the calculated velocity fields with experimental measurements. Also, in order to account for the effects of curvature and rotation, which are present in VAD gap flows, a new experiment is in progress.

## 5. Conclusions

This study aimed to extend our viscosity distribution model for microscale blood flow for significantly higher volume fractions up to 30%. The Einstein–Roscoe equation used for a previous model was found to be inadequate for modeling flows with both high Reynolds numbers and HCT. By integrating results of viscosity measurements and local particle distribution data, the model accounts for blood flows in narrow gaps up to a Reynolds number of 150. This advancement represents a significant step toward the correct numerical characterization of particle-laden flow in the gaps of ventricular assist devices (VADs). The optical analysis of the CFL height showed that it remains constant in the hematocrit range between 5% and 20%. The pressure measurements indicate that the CFL furthermore consistently leads to lower pressure differences up to hematocrits of up to 30%, showing that no substantial change in CFL height occurs in that region. The results show that simulations incorporating the viscosity distribution model align well with the experimental data across all tested hematocrit values. In contrast, simulations based on a single-phase flow assumption revealed significant discrepancies in the calculated pressure losses.

The improved viscosity distribution model can be seamlessly integrated into computational fluid dynamics (CFD) simulations of VADs. Ultimately, this advancement may contribute to more reliable hemocompatibility assessments and support the ongoing optimization and safety of VAD designs.

## Figures and Tables

**Table 1:** Experimentally determined viscosities and densities of porcine blood.

	Plasma	Hct 5%	Hct 10%	Hct 20%	Hct 20% (APTV)	Hct 30%
Density ρ in kg/m^3^	1020.4	1023.61	1026.82	1032	1020.3	1039.3
Dyn. Viscosity μ in mPas	1.31	1.45	1.61	2.07	1.1	2.54

**Table 2:** Wall shear stress (WSS) [Pa] at the centerpoint of the wall surface of the microchannel, calculated from the CFD with single-phase and particle-laden fluid, in comparison to an analytical calculation for single-phase fluid [40].

$\phi_{\mathrm{Bulk}}$	w. Particles	1-Phase	Exp.	Analytical (1-Phase)	Ratio Particles/1-Phase
5%	41.0	45.0	42	44.8	0.911
10%	47.91	51.84	-	52.70	0.924
20%	69.82	85.13	-	86.50	0.820

## Data Availability

The raw data supporting the conclusions of this article will be made available by the authors on request.

## Nomenclature and Abbreviations

The following nomenclature is used in this manuscript:

*A*	Microchannel Cross Section	[m^2^]
$c_{b}$	Bulk Velocity	[m/s]
$c_{p}$	Pressure Coefficient	[-]
$c_{p}/c_{p,0\%}$	Normalized Pressure Coefficient	[-]
$D_{h}$	Hydraulic Diameter	[m]
H,h	Channel Height	[m]
$H_{CFL}$	Height Cell-Free Layer	[m]
*L*	Characteristic Length	[m]
Re	Channel Reynolds Number	[-]
$u_{i},u_{j}$	Velocities	[m/s]
$\dot{V}$	Volume Flow Rate	[m^3^/s]
$x_{i},x_{j}$	Spatial Directions	[m]
$\dot{\gamma }$	Shear Rate	[1/s]
$\mu_{app}$	Apparent Viscosity	[mPas]
$\mu_{STEP_{particles}}$	Viscosity of the Region with Particles	[mPas]
$\mu_{loc}$	Local Viscosity	[mPas]
$\mu_{rheo,\phi \%}$	Bulk Viscosity (from the Rheometer)	[mPas]
$\mu_{\infty }$	Bulk Viscosity	[mPas]
ρ	Density	[kg/m^3^]
$\tau_{ij}$	Shear Stress	[Pa]
$\tau_{w}$	Wall Shear Stress	[Pa]
ϕ	Particle Volume Fraction	[%]
$\phi_{loc}$	Local Particle Distribution	[%]

The following abbreviations are used in this manuscript:

APTV	Astigmatism Particle Tracking Velocimetry
BAF	Blood Analog Fluid
CFD	Computational Fluid Dynamics
CFL	Cell-Free Layer
EXP	Experiment
RBC	Red Blood Cell
VAD	Ventricular Assist Device
WSS	Wall Shear Stress
